# Sulfoxides, Analogues of L-Methionine and L-Cysteine As Pro-Drugs against Gram-Positive and Gram-Negative Bacteria

**Published:** 2015

**Authors:** N. V. Anufrieva, E. A. Morozova, V. V. Kulikova, N. P. Bazhulina, I. V. Manukhov, D. I. Degtev, E. Yu. Gnuchikh, A. N. Rodionov, G. B. Zavilgelsky, T. V. Demidkina

**Affiliations:** Engelhardt Institute of Molecular Biology, Russian Academy of Sciences, Vavilova Str., 32, Moscow, 119991, Russia; State Research Institute of Genetics and Selection of Industrial Microorganisms, 1-st Dorozhniy pr., 1 , Moscow, 117545, Russia

**Keywords:** Pro-drugs, vitamin B6-dependent enzymes, cloning of Clostridium sporogenes methionine γ-lyase gene, alliin, allicin, sulfoxides of amino acids, Gram-positive and Gram-negative bacteria

## Abstract

The problem of resistance to antibiotics requires the development of new classes of broad-spectrum antimicrobial drugs. The concept of pro-drugs allows researchers to look for new approaches to obtain effective drugs with improved pharmacokinetic and pharmacodynamic properties. Thiosulfinates, formed enzymatically from amino acid sulfoxides upon crushing cells of genus *Allium *plants, are known as antimicrobial compounds. The instability and high reactivity of thiosulfinates complicate their use as individual antimicrobial compounds. We propose a pharmacologically complementary pair: an amino acid sulfoxide pro-drug and vitamin B6 – dependent methionine γ-lyase, which metabolizes it in the patient’s body. The enzyme catalyzes the γ- and β-elimination reactions of sulfoxides, analogues of L-methionine and L-cysteine, which leads to the formation of thiosulfinates. In the present work, we cloned the enzyme gene from *Clostridium sporogenes*. Ionic and tautomeric forms of the internal aldimine were determined by lognormal deconvolution of the holoenzyme spectrum and the catalytic parameters of the recombinant enzyme in the γ- and β-elimination reactions of amino acids, and some sulfoxides of amino acids were obtained. For the first time, the possibility of usage of the enzyme for effective conversion of sulfoxides was established and the antimicrobial activity of thiosulfinates against Gram-negative and Gram-positive bacteria *in situ *was shown.

## INTRODUCTION

The development of new antimicrobial agents with a minimal inherent risk of inducing rapid resistance to antibiotics is one of the most pressing issues nowadays. Many potentially effective antimicrobial agents are rapidly degraded in the human body and have high toxicity, preventing their use in the concentrations necessary for treatment. This issue can be resolved through the concept of pro-drugs, compounds that must be metabolized in the body of a patient. This concept has been successfully used in tumor therapy [1].

In the present study, we propose using this approach to create effective antimicrobial therapy using a pharmacological pair of a pro-drug and a biocatalyst metabolizing it. Recently, we have demonstrated that methionine γ-lyase (MGL) [EC 4.4.1.11] from *Citrobacter freundii *catalyzes the β-elimination reaction of a nonprotein amino acid, (±)-S-(2-propenyl)-L-cysteinesulfoxide ((±)-alliin), resulting in 2-propene thiosulfinate (allicin), a natural antibiotic [2].

MGL catalyzes the γ-elimination reaction of L-methionine to produce methylmercaptan, α-ketobutyric acid, and ammonia. The enzyme catalyzes the β-elimination reactions of L-cysteine and its S-substituted derivatives to the corresponding mercaptans, pyruvic acid and ammonia, and the substitution reactions at the C_β_- and C_γ_-atoms of L-cysteine and L-methionine and their analogues [3, 4]:

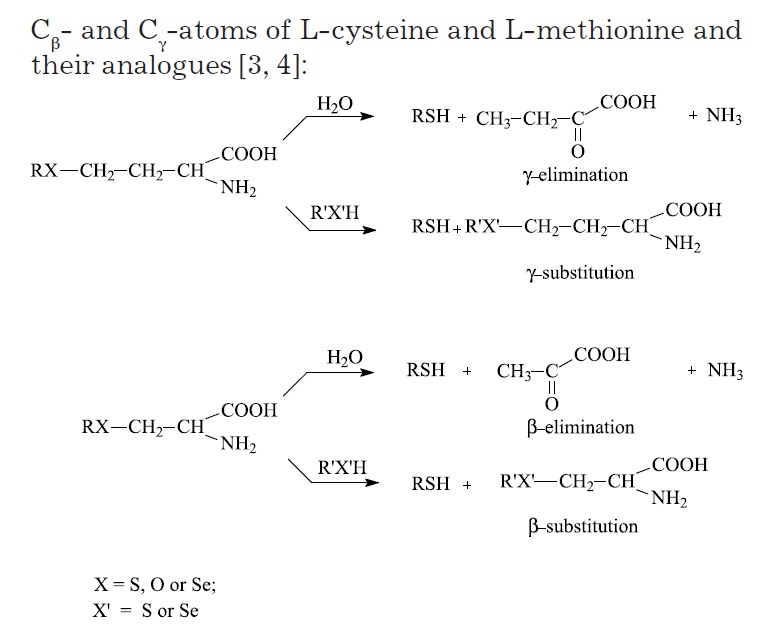



MGL is present in fungi [5], *Arabidopsis thaliana *[6], in various bacteria, including pathogenic *Aeromonas* spp. [7], *Clostridium sporogenes *[8], *Porphyromonas gingivalis *[9], and pathogenic protozoa *Entamoeba histolytica* [10] and *Trichomonas vaginalis *[11]. The enzyme has no counterpart in mammals and, therefore, may be considered as a target in pathogens. This approach was implemented using a suicide substrate of the enzyme. Catalysis of the γ-elimination reaction of trifluoromethionine led to the formation of trifluoromethanethiol, which spontaneously decomposes to thiocarbonyl difluoride, which has an antimicrobial effect on MGL-containing *T. vaginalis *[12], *P. gingivalis* [13], and *E. histolytica *[14]. However, the high toxicity of thiocarbonyl difluoride prohibits the use of trifluoromethionine as an antimicrobial agent.

Allicin, the most well-known antimicrobial and antitumor component of garlic, accounts for about 70% of all thiosulfinates [15] formed by the β-elimination reaction of alliin, which is catalyzed by PLP-dependent alliinase [EC 4.4.1.4] [16] upon crushing of garlic. The antimicrobial action of allicin and other thiosulfinates formed enzymatically during the crushing of plant cells of genus *Allium *is largely due to their ability to oxidize the sulfhydryl groups of proteins/enzymes of bacterial cells, whereas animal cells are partially protected by the presence of glutathione [17]. The antimicrobial, anti-inflammatory, antioxidant, and anticarcinogenic effects of organic sulfo-compounds, cell extracts of garlic and onions [18, 19], have been known since ancient times. However, isolated thiosulfinates are not used in medicine due to their high reactivity and, hence, instability. Only allicin has been studied extensively as an individual biologically active compound, and its antitumor, antioxidant, antibacterial, and antifungal properties have been identified [20-22].

MGL ability to catalyze the γ- and β-elimination reactions of methionine sulfoxide [23] and alliin [2] to produce thiosulfinates allows one to use the concept of prodrugs to develop a new antimicrobial agent, using the substrates of the enzyme, alliin and other sulfoxides, as pro-drugs *in situ *generating thiosulfinates.

Previously, we cloned the *C. sporogenes *gene (*meg*L) encoding MGL with a polyhistidine fragment (His-tag) at the N-terminus of the polypeptide chain and determined some kinetic characteristics of the recombinant enzyme (His-tag MGL). *C. sporogenes *MGL catalyzed the γ-elimination reaction of L-methionine at a faster rate than the enzyme from *C. freundii *[24] and showed higher cytotoxic activity against a number of tumor cells [25].

The cleavage of His-tag by thrombin increases the rate of the physiological substrate cleavage by *C. sporogenes *MGL by 1.5 times. In this study, we cloned the *C. sporogenes *MGL gene without His-tag. The steady-state kinetic parameters of the γ- and β-elimination reactions of a number of well-known substrates and sulfoxides, analogues of cysteine and methionine, and the spectral characteristics of *C. sporogenes *MGL have been determined. The antibacterial activity of mixtures containing MGLs from *C. sporogenes *and *C. freundii *and the sulfoxides of amino acids has been demonstrated in a solid medium. It has been shown that the kinetic parameters of the recombinant PLP-dependent MGL make it possible, in principle, to use the enzyme to convert pro-drugs, sulfoxides of amino acids, to thiosulfinates.

## MATERIALS AND METHODS

**Reagents, enzymes**

The following compounds were used in the study: pyridoxal 5’-phosphate, L-methionine, L-cysteine, L-homocysteine, L-norvaline, L-norleucine, L-α-aminobutyric acid, alliin, S-ethyl-L-cysteine, S-ethyl-L-homocysteine, L-alanine, *O*-acetyl-L-serine, lactate dehydrogenase from rabbit muscle, DTT, NADH, sodium periodate, ethyl bromide (all Sigma, USA); EDTA, protamine sulfate (Serva, USA); lactose (Panreac, Spain); glucose, glycerol, magnesium sulfate, ammonium sulfate, monopotassium phosphate, disodium phosphate (“Reakhim,” Russia); yeast extract, tryptone (Difco, USA); DEAE-Sepharose (GE Healthcare, Sweden); *O*-acetyl-L-homoserine was produced by L-homoserine acetylation as described previously [26]. 2-Nitro-5-thiobenzoic acid was obtained according to [27]. (±)-L-methionine sulfoxide was obtained according to the standard procedure [28]. Synthesis of (±)-S-ethyl-L-cysteine and (±)-S-ethyl-L-homocysteine sulfoxides was performed according to [29-31].

Restriction and ligation reactions were carried out with enzymes from Promega (USA). A “working buffer” with pH 8.0, containing 100 mM potassium phosphate, 0.1 mM PLP, 1 mM DTT, and 1 mM EDTA was used.

*Escherichia coli *strain BL21 (DE3) F- *ompT hsdS*B* gal dcm *(DE3) (Novagen) was used to express the *C. sporogenes *MGL gene. *E. coli *strain K12 AB2463 - a*recA*- derivative of *E. coli *K12, has a F-, *thr-1 leu-6 proA2 his-4 thi-1 argE3 lacY1 galK2 ara-14 xyl-5 mtl-1 tsx-33 rpsL31 supE44*, *recA13 *genotype. It was used for cloning, production, and storage of the plasmid. *C. freundii* strain ATCC 21434 from the American Type Culture Collection (USA) was kindly provided by R. S. Phillips. The *Staphylococcus aureus *strain 015 was kindly provided by Yu. F. Belyi. The plasmid with D-2-hydroxyisocaproate dehydrogenase was kindly provided by K. Muratore.

**Cloning of the *C. sporogenes *MGL gene**

The pET28a-*meg*L_sporog plasmid was constructed based on the pET28a plasmid, containing the* C. sporogenes meg*L gene with a polyhistidine fragment (His-tag) and designated as pET28a::megL_s_ HT [24]. The amplicon (*meg*L_sporog), containing the* meg*L gene without His-tag, was obtained by PCR. pET28a plasmid carrying *meg*L with His-tag was used as a template. The primers included the NcoI restriction site (underlines): *meg*L_sporog:5’-CGCGCGGCAGCC**CCATGG**AGAA- 3’(forward), *meg*L_ sporog:5’-CCGGATCTCAGTGGTGGTGGTG-3’ (reverse).

*Meg*L_sporog amplicon was cloned in the pET28a vector by the NcoI and EcoRI sites in the *recA*- *E. coli* strain AB2463. The cloning was controlled by sequencing the insert. Transformation was carried out using the *E. coli *strain BL21 (DE3).

**Biomass growth and enzyme purification**

Cells of *E. coli *BL21 (DE3), containing the MGL gene without His-tag in the pET28a *meg*L_sporog plasmid, were grown in the “inducing” medium [32] at 37 °C with stirring (180 rpm) for 24 hours. The cells were collected by centrifugation and stored at -80 °C. The cells were destroyed and purified from nucleic acids as described previously [33]. Further purification was carried out by ion exchange chromatography on a column with DEAE-Sepharose equilibrated with the working buffer. The column was pre-washed with the working buffer containing 100 mM KCl. The enzyme was eluted with the working buffer containing 500 mM KCl, concentrated and dialyzed against the working buffer. The purity of the preparation was checked by polyacrylamide gel electrophoresis under denaturing conditions according to Laemmli [34]. The concentration of the purified preparations was determined using a A_1%_^278^coefficient of 0.8 [23].

**Assay of the enzyme activity and steadystate kinetics parameters**

MGL activity during the purification was assayed in the γ- and β-elimination reactions by measuring the reduction of NADH absorption at 340 nm (ε = 6220 M^-1^cm^-1^) at 30 °C to estimate the rate of keto acids formation in the conjugation reaction with D-2-hydroxyisocaproate dehydrogenase (the γ-elimination reaction) or lactate dehydrogenase (the β-elimination reaction). The reaction mixtures contained the working buffer, 0.2 mM NADH, 10 units of lactate dehydrogenase or 70 μg of D-2-hydroxyisocaproate dehydrogenase, 30 mM S-ethyl-L-cysteine, or 30 mM L-methionine. One unit of enzyme activity was defined as the amount of the enzyme that catalyzes the formation of 1.0 μM/min of pyruvate (or α-ketobutyrate). The specific activity of 95% pure enzyme preparations was 26.8 units/mg for the γ-elimination reaction of L-methionine and 8.32 unit/mg for the β-elimination reaction of S-ethyl-L-cysteine.

Steady-state kinetic parameters for the γ- and β-elimination reactions were measured in the same manner by varying the substrates concentrations. The obtained data were processed according to the Michaelis- Menten equation using the EnzFitter software. Calculations were based on the molecular weight of an enzyme subunit of 43 kDa. Inhibition of the γ-elimination reaction of L-methionine by various amino acids was studied under the conditions described above by varying the concentrations of substrates and inhibitors in the reaction mixture. The values of inhibition constants were determined using the EnzFitter software. The data were processed in Dixon coordinates [35].

**Spectral studies**

The absorption spectrum of holoenzyme was recorded at 25 °C on a Cary-50 spectrophotometer (Varian, USA) in the working buffer without PLP. The enzyme concentration was 1.036 mg/mL.

**Antimicrobial activity of drugs**

Overnight cultures of *C. freundii *and *S. aureus *grown in a Luria-Bertani medium (LB-medium) at 37 °C were diluted 100-fold in a LB-medium and grown at 37 °C with constant stirring to an optical density of 0.2–0.3 at 600 nm. The bacterial cultures were plated on solid- medium dishes (LB-agar). Mixtures of MGLs from different sources and sulfoxides of amino acids pre-incubated at room temperature for 1 hour were applied to 12 mm filter paper disks placed on the dishes. The concentrations of MGLs from *C. sporogenes *and *C. freundii* and sulfoxides were 10 and 2.5 mg/mL, respectively. The dishes were incubated for 24 hours at 37 °C, and inhibition zones were then measured. The control solutions of the enzymes and the sulfoxides mixtures retained their antibacterial activity for 2 weeks.

**Determination of allicin**

Allicin, produced in the mixtures containing MGL and alliin, was determined in a reaction with 2-nitro- 5-thiobenzoic acid. The mixture of MGL and alliin was added to 1 mL of 0.1 mM 2-nitro-5-thiobenzoic acid in a 100 mM potassium-phosphate buffer containing 0.2 mM PLP, pH 8.0. The mixture was incubated for 30 min at room temperature. Allicin molar concentration was calculated by the decrease in absorbance at 412 nm using a molar absorption coefficient of 2-nitro-5-thiobenzoic acid at 412 nm of 28,300 M^-1^cm^-1^ [27].

## RESULTS AND DISCUSSION

**Kinetic parameters of the β- and γ-elimination reactions**

Previously [25], we showed that cleavage of His-tag from *C. sporogenes *MGL by thrombin leads to a 1.5- fold increase in the activity of the enzyme in the physiological reaction with L-methionine. In this work, we have determined the parameters of steady-state kinetics of *C. sporogenes *MGL without His-tag in the γ-elimination reactions of five substrates (L-methionine, L-methionine sulfoxide, S-ethyl-L-homocysteine, S-ethyl-L-homocysteine sulfoxide and *O*-acetyl-L-homoserine) and in the β-elimination reactions of four substrates (S-ethyl-L-cysteine, S-ethyl-L-cysteine sulfoxide, *O*-acetyl-L-serine and alliin). *Table 1* summarizes the parameters for MGL from *C. sporogenes*, for MGLs derived from two other bacterial sources, and *C. sporogenes *His-tag MGL.

The *k*_cat_ values for *C. sporogenes *MGL in the γ-elimination reactions of three substrates, L-methionine, S-ethyl-L-homocysteine, and L-methionine sulfoxide, were 2–3 times higher than for *C. sporogenes *Histag MGL. *K*_M_ values for the first two substrates were close, and the *K*_M_ value for L-methionine sulfoxide was slightly higher than that for His-tag MGL.

The presence of the His-tag fragment does not affect the kinetic parameters of the β-elimination reaction of S-ethyl-L-cysteine, and *K*_M_ and *k*_cat_ values for MGL are almost identical to those for His-tag MGL. In the γ- and β-elimination reactions, the elimination of the side-chain groups of the substrates is catalyzed by different acid groups of the enzyme. Presumably, in the case of the β-elimination reaction catalyzed by PLP-dependent lyases, this group is the side group of the lysine residue (Lys210 in *C. freundii *MGL) which binds the coenzyme [36]. In PLP-dependent γ-elimination and γ-replacement reactions, this role is attributed to the conservative tyrosine residue (Tyr113 in* C. freundii *MGL) involved in the stacking interaction with the coenzyme ring [36]. This assumption is confirmed by the data obtained for the mutant form of* Pseudomonas putida *MGL, in which Tyr114 is replaced with Phe [37]. It has also been shown that the acid/base properties of Tyr113 in *C. freundii *MGL are regulated by the Cys115/Tyr113/Arg60 triad [2]. Arg60 is located in the mobile N-terminal loop of the enzyme, and the nitrogen atom of the guanidine group is positioned within a hydrogen-bond distance from the hydroxyl group of Tyr113 in the three-dimensional structure of the holoenzyme [38], the structures of MGL com plexes with amino acids modeling the Michaelis complex [39], and in the spatial structure of the external aldimine of the enzyme with glycine [40]. The His-tag fragment may affect the conformation of the N-terminal loop and, therefore, the relative arrangement of the hydroxyl group of Tyr113 and the guanidine group of Arg60, which, in turn, may affect the p*K*_a_ value of the hydroxyl group of Tyr113. That may explain the increase in the γ-eliminating activity of *C. sporogenes* MGL compared with His-tag MGL.

Comparison of the enzymes from three bacterial sources (*Table 1*), *P. putida*, *C. freundii, *and *C. sporogenes*, showed that their affinity for both the physiological substrate and its analogues are almost equal. The efficiency of catalysis in the reaction γ-elimination of L-methionine for *C. sporogene *and *P. putida* MGLs is close, and the *k*_cat_/*K*_M_ value for *C. freundii* MGL is somewhat lower. The kinetic parameters of the β-elimination reaction of S-ethyl-L-cysteine are very similar for the three enzymes.

**Table 1:** Kinetic parameters of the γ- and β-elimination reactions*

Substrate	C. sporogenes MGL			C. sporogenes His-tag MGL**			C. freundii MGL***			P. putida MGL****		
	k_cat_, s^-1^	K_M_, mM	k_cat_/K_M_ M^-1^s^-1^	k_cat_, s^-1^	K_M_, mM	k_cat_/K_M_ M^-1^s^-1^	k_cat_, s^-1^	K_M_, mM	k_cat_/K_M_ M^-1^s^-1^	k_cat_, (s^-1^)	K_M_, mM	k_cat_/K_M_ M^-1^s^-1^
L-Met	21.61	0.60	3.60 × 10^4^	9.86	0.43	2.28 × 10^4^	6.2	0.7	8.85 × 10^3^	48.6	0.90	5.4 × 10^4^
(±)-L-MetO	21.66	11.39	1.90 × 10^3^	8.59	7.89	1.09 × 10^3^	8.12	4.65	1.75 × 10^3^	-	-	-
S-Et-L-Hcy	21.31	0.24	8.87 × 10^4^	7.05	0.27	2.54 × 10^4^	6.78	0.54	1.25 × 10^4^	33.4	0.27	1.23 × 10^5^
(±)-S-Et-L-HcyO	0.48	0.60	8.0 × 10^2^	-	-	-	-	-	-	-	-	-
O-Ac-L-Hse	37.26	3.18	1.17 × 10^4^	-	-	-	2.1	2.91	2.91 7.21 × 10^2^	78.0	2.22	3.51 × 10^4^
S-Et-L-Cys	6.53	0.43	1.52 × 10^4^	6.3	0.358	1.76 × 10^4^	5.03	0.17	2.96 × 10^4^	5.79	0.48	1.21 × 10^4^
(±)-S-Et-L-CysO	1.39	0.33	4.21 × 10^3^	-	-	-	-	-	-	-	-	-
O-Ac-L-Ser	5.31	8.01	6.6 × 10	-	-	-	2.13	4.28	4.98 × 10^2^	-	-	-
(±)-Alliin	11.43	1.43	7.99 × 10^3^	-	-	-	5.9	4.7	1.26 × 10^3^	-	-	-

*The error did not exceed 10%.

**Data from [25].

***Data from [2, 23, 33].

****Data from [37].

*C. sporogenes *MGL catalyzes the γ-elimination reaction of L-methionine sulfoxide with a catalytic efficiency which is an order of magnitude higher than that in the γ-elimination reaction of S-ethyl-L-homocysteine sulfoxide. The rate of the β-elimination reaction of S-ethyl- L-cysteine sulfoxide, catalyzed by the enzyme, is 15 times lower than the rate of the γ-elimination reaction of L-methionine sulfoxide, but due to the greater affinity of *C. sporogenes *MGL to this substrate, the overall catalytic efficiency is virtually the same. Among the reactions with amino acids sulfoxides, the enzyme most effectively catalyzes the β-elimination reaction of alliin.

The enzyme from *C. sporogenes *catalyzes the γ-elimination reaction of L-methionine sulfoxide more effectively than *C. freundii *MGL (*k*_cat_ value is 2.5 times higher). The rate of alliin cleavage by *C. sporogenes* MGL is almost 2 times higher than that of the enzyme from *C. freundii*, the substrate affinity is 3 times higher, and the efficiency of catalysis is 6.3 times higher.

Amino acids with a linear side chain inhibited the γ-elimination reaction of L-methionine competitively. *Table 2* shows the inhibition constants for *C. sporogenes*,* C. freundii, *and *P. putida *MGLs. All of these enzymes demonstrate an increase in binding with an increase in the number of methylene groups in amino acids with linear side chains, which can be attributed to the hydrophobic nature of the active site of the enzyme from* P. putida *[41] and *C. freundii *[38]. The significant increase in the affinity of the enzyme from the three sources then switching from L-norvaline to L-norleucine and close values of *K*i for L-norleucine and *K*M for L-methionine and S-ethyl-L-cysteine may be attributed to the presence of a “pocket” for the amino acid methyl group in the MGL active site.

**Table 2:** Inhibition of the γ-elimination reaction of L-methionine*

Amino acid	K_i_, mM		
	C. freundii**	C. sporogenes	P. putida***
L-Ala	3.4	1.5	5.1
L-Abu	8.3	2.0	8.4
L-Nva	4.7	1.9	3.0
L-Nle	0.6	0.37	0.5

* The error did not exceed 10%.

** Data from [23].

*** Data from [43].

**Spectral characteristics of the enzyme**

**Fig. 1:**
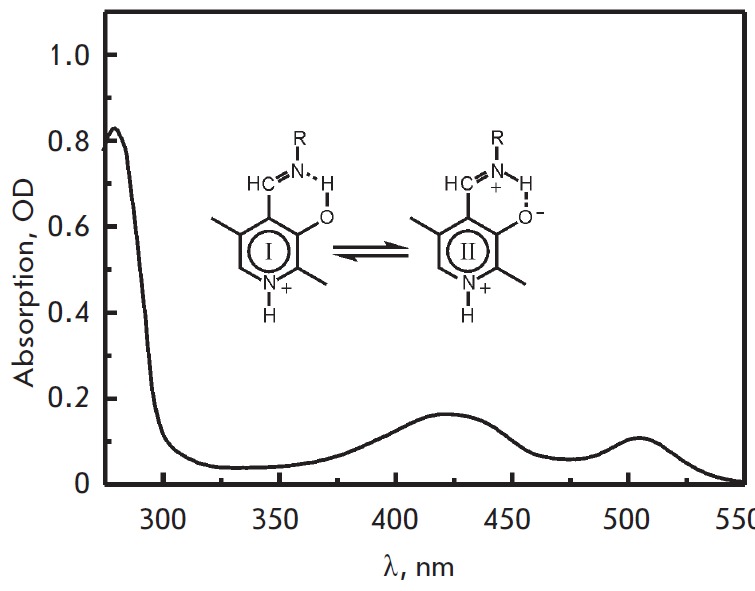
Absorption spectrum of the holoenzyme* C. sporogenes *MGL

The absorption spectrum of *C. sporogenes *MGL holoenzyme (*Fig. 1*) at pH 8.0 is similar to the spectrum of* C. freundii *MGL [23], with a predominant absorption band of the ketoenamine form of the internal aldimine in the region 422-425 nm (*Fig. 1, structure II*). Just like* C. sporogenes *His-tag MGL [24], the spectrum contains an intense absorption band with a maximum in the region 502-505 nm, which is attributed to a quinonoid intermediate in the spectra of PLP-dependent enzyme complexes with amino acids and model compounds [42].

Deconvolution of the holoenzyme spectrum in the region 300–500 nm using lognormal curves was performed according to [23]. *Table 3* shows the parameters of the absorption bands obtained after deconvolution. In addition to the ketoenamine form, the internal aldimine (*Fig. 1, structure II*, ε = 10410 M-1s- 1) is represented by minor structures, enol tautomer (*Fig. 1, structure I*), and two ketoenamine conformers with the aldimine bond perpendicular to the plane of the coenzyme ring (absorption in the region of 380 nm) and with the aldimine bond partly removed from the plane of the ring but retaining its coupling with π-electrons of the cofactor and a hydrogen bond between aldimine nitrogen and the 3’-oxygroup of PLP (absorption in the region 327–328 nm). The ionic form of the internal aldimine and tautomeric equilibrium are almost the same as those for *C. freundii *MGL. The absorption in the region 502–505 nm requires further investigation.

**Table 3:** Parameters of the absorption spectrum bands of the internal aldimine C. sporogenes MGL

Structure	E, eV	ν × 10^-3^, cm^-1^	λ, nm	ε × 10-3, M^-1^cm^-1^	W × 10^-3^, cm^-1^	ρ	f	n, %
II^1^	2.92	23.53	425.0	10.46	3.58	1.58	0.22	64.7
II	3.24	26.15	382.4	7.76	4.00	1.37	0.02	7.5
I	3.63	29.28	341.5	9.44	3.65	1.23	0.03	10.0
II^┴^	3.79	30.56	327.2	10.27	3.47	1.29	0.01	5.6
II^2*^	4.28	34.55	289.4	5.98	5.06	1.20	0.18	
*	4.46	35.99	277.9	6.70	4.70	1.50	0.26	

E, electron transition energy; ν, wave number; λ, wavelength; ε, molar absorption coefficient; W, half–width; ρ, asymmetry; f, oscillator force; n, contents of tautomers and conformers. The content of PLP in the enzyme is 87.8%.

* Experimental information about these bands is insufficient. Above-line indices (1, 2) correspond to the first and second electron transitions of structure II. Above-line indices (^┴^,^ < ^) correspond to two conformers of structure II (the conformer with the aldimine group in the plane perpendicular to the pyridine cycle plane and the conformer with the aldimine bond released from the coenzyme ring plane but with retained coupling and a hydrogen bond between the aldimine nitrogen atom and the coenzyme 3’-oxygroup).

**Antimicrobial activity of mixtures of* C. freundii *and *C. sporogenes *MGLs with sulfoxides of amino acids**

**Fig. 2:**
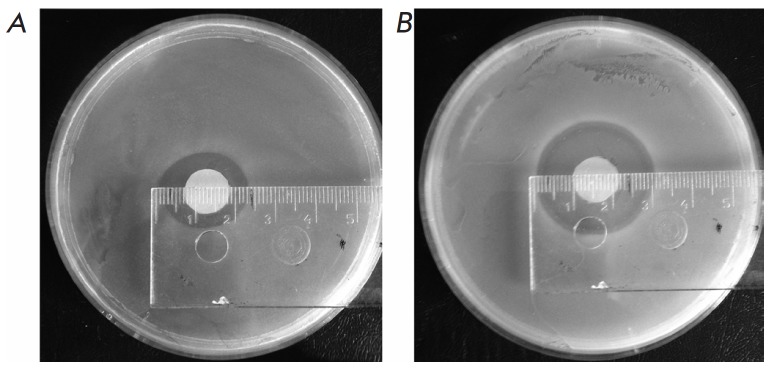
Diffusion in agar by the Kirby-Bauer method [44]. The mixture of *C. sporogenes *MGL (10 mg/ml) and alliin (2.5 mg/ml) in 100 mM potassium phosphate-buffer was applied on the – A) cell culture of *C. freundii*, B) cell culture of *S. aureus*

The antibacterial activity of mixtures of MGLs from two sources and sulfoxides of amino acids was assessed using bacterial cultures of Gram-positive* S. aureus *and Gram-negative *C. freundii* (*Table 4*). All mixtures showed a bacteriostatic effect against Gram-positive and Gram-negative bacteria. The most significant effect was observed for the culture of *S. aureus* (*Fig. 2*). The bacteriostatic effect was comparable to the inhibition of bacterial cell growth by kanamycin. The inhibition zones of kanamycin (0.05 mg) and a mixture comprising 0.04 mg of allicin in the *C. freundii *culture amounted to 314 and 346 mm2, respectively.

Therefore, the data obtained show that the recombinant enzyme effectively catalyzes the conversion of amino acids sulfoxides into thiosulfinates. This suggests that a pharmacological pair of MGL and a sulfoxide can ensure production of thiosulfinates in the amounts necessary for therapeutic purposes.

**Table 4:** Inhibition of cell culture by mixtures containing MGL and sulfoxides of amino acids

Amino acid sulfoxide	Inhibition zone, mm^2^			
	C. freundii MGL		C. sporogenes MGL	
	C. freundii	S. aureus	C. freundii	S. aureus
(±)-Alliin	380	754	254	754
(±)-L-MetO	452	491	177	227
(±)-S-Et-L-CysO	314	491	254	314
(±)-S-Et-L-HcyO	254	415	227	227

## CONCLUSIONS

MGL catalyzes the γ- and β-elimination reactions of sulfoxides, analogues of methionine and cysteine, with a catalytic efficiency comparable to the efficiency of the γ- and β-elimination reactions of these amino acids.

Using a solid medium, we have demonstrated that mixtures of sulfoxides and MGL are promising as antimicrobial agents against Gram-positive and Gram-negative bacteria *in situ*.

The strongest bacteriostatic effect for the mixture of amino acids sulfoxides and MGL have been observed for Gram-positive bacteria *S. aureus*, and the bacteriostatic effect of allicin produced *in situ *is comparable with the effect of kanamycin.

## Abbreviations

PLP: pyridoxal 5’-phosphate
MGL: methionine γ-lyase
His-tag: poly-histidine fragment
His-tag MGL: methionine γ-lyase with poly-histidine fragment
megL: gene encoding of MGL in Clostridium sporogenes
DTT: dithiothreitol
NADH: reduced form of β-nicotinamide adenine dinucleotide
EDTA: ethylenediaminetetraacetic acid
